# Experimental database of optical properties of organic compounds

**DOI:** 10.1038/s41597-020-00634-8

**Published:** 2020-09-08

**Authors:** Joonyoung F. Joung, Minhi Han, Minseok Jeong, Sungnam Park

**Affiliations:** Department of Chemistry and Research Institute for Natural Science, Korea University, Seoul, 02841 Korea

**Keywords:** Fluorescence spectroscopy, Excited states, Cheminformatics, Chemical physics

## Abstract

Experimental databases on the optical properties of organic chromophores are important for the implementation of data-driven chemistry using machine learning. Herein, we present a series of experimental data including various optical properties such as the first absorption and emission maximum wavelengths and their bandwidths (full width at half maximum), extinction coefficient, photoluminescence quantum yield, and fluorescence lifetime. A database of 20,236 data points was developed by collecting the optical properties of organic compounds already reported in the literature. A dataset of 7,016 unique organic chromophores in 365 solvents or in solid state is available in CSV format.

## Abstract (summary)

**Table:** 

Measurement(s)	absorption wavelength • emission wavelength • fluorescence lifetime • bandwidth • extinction coefficient • electronic absorption spectrum • fluorescence spectrum • optical property of organic compound
Technology Type(s)	ultraviolet-visible spectrophotometry • spectrofluorimeter • time-resolved fluorescence spectroscopy • digital curation
Factor Type(s)	organic molecule • solvent

Machine-accessible metadata file describing the reported data: 10.6084/m9.figshare.12808424

## Background & Summary

Organic chromophores used in optoelectronics, organic light emitting diodes (OLEDs), staining, fluorescent dyes, and bioimaging dyes, have been steadily developed. Therefore, it would be useful to reliably and quickly predict the optical properties of newly designed organic chromophores prior to their synthesis. Theoretical calculations based on ab initio and density functional theory methods have been extensively used to characterize the optical properties of newly designed organic chromophores. However, such theoretical calculations require high computational costs. Therefore, data-driven sciences based on machine learning have emerged as a promising alternative method and have been applied in many research areas^1–3^. However, databases are a prerequisite for data-driven sciences based on machine learning. Thus, databases for specific applications need to be available or collected.

The optical properties, such as absorption and emission maximum wavelengths and their bandwidths, extinction coefficient, photoluminescence quantum yield (PLQY), and lifetime, are important factors in characterizing organic chromophores. Therefore, databases on optical properties can be used to model the quantitative structure–property relationship for designing new organic chromophores with desired optical properties. Recently, the absorption peaks and extinction coefficients of small organic molecules have already been obtained using quantum chemical calculations and have been used for machine learning^4–6^. In addition, Beard *et al*. have reported the datasets of experimental and computational ultraviolet–visible (UV–Vis) absorption spectra^7^. However, no databases are currently available for the experimental absorption, emission, and fluorescence properties of organic chromophores.

**Fig. 1:**
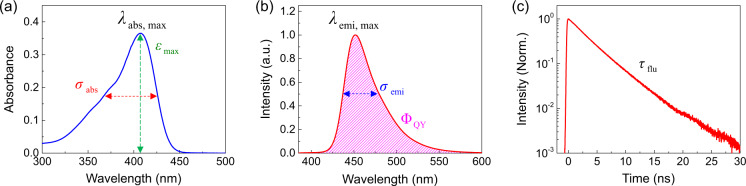
(**a**) Absorption spectrum displaying *λ*_abs, max_, *σ*_abs_ (in FWHM), and *ε*_max_. (**b**) Emission spectrum displaying *λ*_emi, max_, *σ*_emi_ (in FWHM), and Φ_QY_. (**c**) Time-resolved fluorescence (TRF) signal and *τ*_flu_.

As illustrated in Fig. 1, the absorption properties of organic chromophores are characterized by the first maximum absorption wavelength (*λ*_abs, max_), bandwidth (*σ*_abs_), and extinction coefficient (*ε*_max_) (Fig. 1a), which are important parameters for the design of chromophores for specific applications in various research fields such as photovoltaics, dyes, and optical filters. Similarly, the emission and fluorescent properties, which are characterized by the maximum emission wavelength (*λ*_emi, max_), bandwidth (*σ*_emi_), PLQY (Φ_QY_), and excited state lifetime (*τ*) (Fig. 1b, c), are essential for the development of emitters in OLEDs, fluorescent bioimaging dyes, and fluorescent sensors. In this study, we present a reliable and high-quality database of the optical properties of organic compounds that can be used for various purposes in diverse research fields.

## Methods

A total of 1,358 articles containing organic compounds were downloaded from journals of Nature Research, American Chemical Society, Royal Society of Chemistry, Springer, and Elsevier by exploring keywords such as fluorescence, luminescence, emission, OLED, fluorescence lifetime, or PLQY.

In our database, the organic compounds and solvent molecules are limited to a maximum number of 150 atoms (except hydrogen atoms) consisting of C, N, O, S, F, Cl, Br, I, Se, Te, Si, P, B, Sn, and Ge. Binary or ternary solvent systems are not included in our database. Data points in the solid state include one component systems (either amorphous or crystalline) and the solid solution such as dopant (chromophore) – host (solvent) systems in our database.

All the optical properties in our database are based on the absorption and emission spectra reported in the originally published papers. To extract the optical properties, the absorption and emission spectra were carefully examined to exclude unreliable experimental results. In the case of collecting the extinction coefficients, absorption maximum, and absorption bandwidth, the background corrected absorption spectra in the dynamic range (typically, absorbance < 2) were selected. Similarly, for collecting the emission maximum, bandwidth, and quantum yield, the properly measured emission spectra were carefully selected. The Φ_QY_ values exceeding 1 were not included. In the absorption spectrum of a given molecule, the first absorption peak was selected and its *λ*_abs, max_, *σ*_abs_ (in full width at half maximum (FWHM)), and *ε*_max_ values were obtained. Likewise, the *λ*_emi, max_ and *σ*_emi_ (in FWHM) values were obtained from the emission (or fluorescence) spectra. The bandwidths (*σ*_abs_ and *σ*_emi_) were reported in cm^−1^ or nm as provided in the published papers.

Furthermore, the PLQY (Φ_QY_) measured in degassed media was preferentially collected, if reported. Otherwise, the PLQY measured in the air was collected. The fluorescence (or excited state) lifetimes (*τ*) measured by time-resolved fluorescence (TRF) experiments were also collected. In the case that the TRF signal was fit using a multi-exponential function [$S\left(t\right)=\sum _{i}\,{A}_{i}{\rm{\exp }}(-t/{\tau }_{i})$] where *A*_i_ and *τ*_i_ is the amplitude and time constant, the average lifetime [$\tau =\sum _{i}\,{A}_{i}{\tau }_{i}/\sum _{i}\,{A}_{i}$] was obtained and recorded. The molecular structures are reported in the canonicalized simplified molecular input line entry system (SMILES)^8–11^. For the optical properties, a pair of chromophore and solvent are provided, whereas for solid states, the chromophore is used as both chromophore and solvent. Moreover, for chromophores in a solid matrix, the solid matrix is used as the solvent.

## Data Records

The developed database is available at figshare^12^ and its format is described in Table 1. The database comprises 20,236 combinations of 7,016 chromophores in 365 solvents and 17 solid matrices (or host) or solid states. Furthermore, the SMILES strings of the chromophores and solvents are provided and they indicate their molecular structures. All experimental data from the literature are presented with the corresponding reference, and each digital object identifier (DOI) is also reported. An example of benzene in cyclohexane is presented in Table 2. The data that are not reported in the references are indicated as NaN (not a number).

**Table 1:** Description of the database.

No.	Column name	Unit	Data type	Description
1	Tag	—	Float	The numbering of data points
2	Chromophore	—	String	SMILES of chromophore structure
3	Solvent	—	String	SMILES of solvent structure
4	Absorption max (nm)	nm	Float	Maximum absorption wavelength, ${\lambda }_{{\rm{abs,max}}}$
5	Emission max (nm)	nm	Float	Maximum emission wavelength, ${\lambda }_{{\rm{emi,max}}}$
6	Lifetime (ns)	ns	Float	Fluorescence lifetime, ${\tau }_{{\rm{flu}}}$
7	Quantum yield	—	Float	Photoluminescence quantum yield, ${\Phi }_{{\rm{QY}}}$
8	log(e/mol-1 dm3 cm-1)	—	Float	Extinction coefficient at ${\lambda }_{{\rm{abs,max}}}$, ${\log }_{10}({\varepsilon }_{{\rm{\max }}})$
9	abs FWHM (cm-1)	cm^−1^	Float	Absorption bandwidth (FWHM), ${\sigma }_{{\rm{abs}}}$
10	emi FWHM (cm-1)	cm^−1^	Float	Emission bandwidth (FWHM), ${\sigma }_{{\rm{emi}}}$
11	abs FWHM (nm)	nm	Float	Absorption bandwidth (FWHM), ${\sigma }_{{\rm{abs}}}$
12	emi FWHM (nm)	nm	Float	Emission bandwidth (FWHM), ${\sigma }_{{\rm{emi}}}$
13	Molecular weight (g mol-1)	g mol^−1^	Float	Molecular weight of chromophore
14	Reference		String	Source document DOI

**Table 2:** Optical properties of benzene in cyclohexane.

Tag	4461
Chromophore	c1ccccc1
Solvent	C1CCCCC1
Absorption max (nm)	252.5253
Emission max (nm)	287.3563
Lifetime (ns)	60
Quantum yield	0.14
log(e/mol-1 dm3 cm-1)	NaN
abs FWHM (cm-1)	NaN
emi FWHM (cm-1)	NaN
abs FWHM (nm)	NaN
emi FWHM (nm)	NaN
Molecular weight (g mol-1)	78.11364
Reference	10.1016/S1386-1425(02)00207-X

## Technical Validation

The main purpose of our database is to provide the optical properties of chromophores to the scientific and industrial communities with high quality and reliability.

The validation of the data we collected relies on the validation of peer-reviewed articles. To reduce the potential errors, we built our database in the following procedure. Two people, who had sufficient background in spectroscopic measurements, separately collected the optical properties from the published papers. The third person cross-checked these two datasets and added them to the database. In addition, the outliers such as *λ*_abs, max_ (*λ*_emi, max_) > 950 nm or <200 nm, *λ*_abs, max_ > *λ*_emi, max_, *σ*_abs_ or *σ*_emi_ > 7000 cm^−1^, *τ*_flu_ < 0.1 ns, and log_10_(*ε*_max_) < 2.5, were double-checked. Therefore, all the values in the final version of our database were carefully checked with those values and the spectra in the originally published papers.

**Fig. 2:**
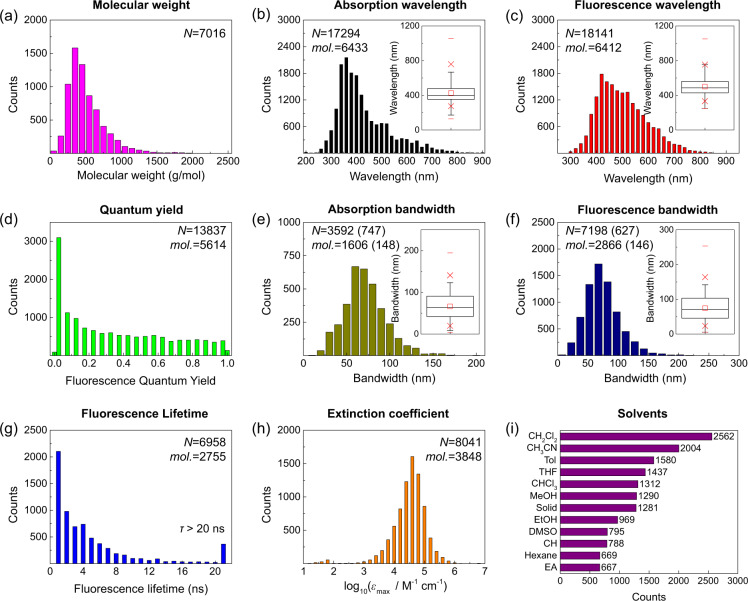
Histograms of (**a**) the molecular weight of chromophores, (**b**) *λ*_abs, max_, (**c**) *λ*_emi, max_, (**d**) Φ_QY_, (**e**) *σ*_abs_, (**f**) *σ*_emi_, (**g**) *τ*_flu_, (**h**) log_10_(*ε*_max_), and (**i**) solvents (CH_2_Cl_2_: dichloromethane, CH_3_CN: acetonitrile, Tol: toluene, THF: tetrahydrofuran, CHCl_3_: chloroform, MeOH: methanol, EtOH: ethanol, DMSO: dimethyl sulfoxide, CH: cyclohexane, and EA: ethyl acetate). The number of data points and the unique molecules are included in each graph. The box plots are drawn inside (**b**,**c**,**e**,**f**). The numbers in the parenthesis in (**e**,**f**) represent the number of the bandwidths reported in cm^−1^ and the number of the corresponding molecules, respectively.

A summary of the developed database is provided in Fig. 2. Among the 7,016 chromophores that can be found in our database, 95.2% have molecular weights lower than 1000 g/mol. Moreover, the chromophores contain diverse core structures such as pyrene, coumarin, perylene, porphyrin, boron-dipyrromethene (BODIPY), stilbene, azobenzene, and so on. In addition, the chromophores with molecular weight higher than 1000 g/mol generally comprise long alkyl chains or sugar units, which are introduced to improve the solubility without affecting the optical properties.

The histograms of *λ*_abs, max_ and *λ*_emi, max_ in Fig. 2b,c are divided into bins with a 20-nm width, covering a wide range of *λ*_abs, max_ and *λ*_emi, max_. For example, 63% of *λ*_abs, max_ and 88% of *λ*_emi, max_ are in the visible range (380–700 nm), whereas more than 93% of the chromophores can absorb sunlight (310–750 nm), indicating their potential use as dyes and light harvesting molecules. Furthermore, our database contains fluorophores covering a wide range of emission wavelengths from UV to near infrared (NIR), which are applicable to OLEDs, fluorescence imaging dyes, and fluorescence sensors. In addition, chromophores with various functional groups^13,14^ and a chromophore in various solvents^15,16^ are included so that the effects of the functional groups and the solvents (solvatochromism) on the optical properties of the chromophores are well documented.

The histogram of the collected Φ_QY_ values is also divided into bins with a width of 0.05 (Fig. 2d). The standards for PLQYs, such as quinine sulfate and rhodamine 6G in solution, are also included^17^. Among the obtained QY data, the Φ_QY_ of 91 data points is 0, and that of 137 data points is 1, whereas for approximately 23% of the QY data, the Φ_QY_ is less than 0.05. Furthermore, the PLQYs of 803 samples in solid state were obtained mainly from OLED molecules^18,19^. In addition, molecules exhibiting aggregation induced emission were also collected for our database^20,21^.

Figure 2e,f display the *σ*_abs_ and *σ*_emi_ values that were extracted from the absorption and emission spectra of over 1,600 and 2,800 molecules, respectively. Our database contains 3292 and 7198 data points of *σ*_abs_ and *σ*_emi_ reported in nm and 747 and 627 data points of *σ*_abs_ and *σ*_emi_ reported in cm^−1^. *σ*_abs_ and *σ*_emi_ values were barely reported in the published papers when compared with other optical properties. Most of *σ*_abs_ and *σ*_emi_ values in nm were extracted directly from the absorption and emission spectra reported in the originally published papers.

The standards for the fluorescence lifetime (*τ*_flu_) values reported by Boens *et al*.^22^ as well as other *τ*_flu_ measurements were also included in our database. The histogram of the collected *τ*_flu_ is divided into bins with a width of 1 ns (Fig. 2g), indicating that approximately 5% of the *τ*_flu_ values is longer than 20 ns.

The *ε*_max_ values at *λ*_abs, max_ are recorded in log_10_(*ε*_max_) and their distribution in Fig. 2h is shown in the histogram which is divided into bins with a width of 0.2. In our database, most of the *ε*_max_ values are in the range of 10^3^–10^6^ mol^−1^ dm^3^ cm^−1^ (mol^−1^ L cm^−1^). Note that the product of Φ_QY_ and *ε*_max_ is proportional to the brightness, which is the fluorescence intensity per fluorophore. In addition, the number of data points simultaneously exhibiting Φ_QY_ and *ε*_max_ is 6,663, which can be used to estimate the brightness.

Finally, the optical properties of chromophores are solvent-dependent. In our database, 365 solvents are included. Among the 12 most common solvents presented in Fig. 2i, dichloromethane is the most frequently used. Moreover, alkanes with a number of carbon atoms ranging from 2 (1,1-dichloroethane) to 16 (1-chlorohexadecane) and 99 alcohols with one (methanol) to 12 (dodecanol) carbon atoms are reported as solvents. In addition, solid solutions and host molecules, such as 4,4ʹ-bis(carbazol-9-yl)biphenyl, bis[2-(diphenylphosphino)phenyl] ether oxide (DPEPO), and 1,3-bis(N-carbazolyl)benzene (mCP), are included in our database.

### Estimation of experimental uncertainties of the optical properties in the database

The optical properties of organic compounds were collected from the published peer-reviewed papers. In most original papers, the experimental uncertainties in seven optical properties were not reported. In addition, the experimental conditions were different when the optical properties were measured. Therefore, it is very difficult to accurately estimate the experimental uncertainties. However, the experimental uncertainties of the optical properties are roughly estimated in the following way.

#### Experimental uncertainty of λ_abs, max_ and λ_emi, max_

Most of UV-visible absorption and emission spectra reported in the published papers were measured by the spectrophotometers and spectrofluorometers available from Agilent, Ocean optics, Hitachi, and JASCO. Including them, the typical and modern absorption and emission spectrometers have a wavelength resolution of less than 1 nm. The maximum wavelengths (*λ*_abs, max_ and *λ*_emi, max_) of absorption and emission spectra can be readily determined within an experimental error of 1 nm. Therefore, the experimental uncertainty of *λ*_abs, max_ and *λ*_emi, max_ is estimated to be less than 1 nm.

#### Experimental uncertainty of σ_abs_ and σ_emi_

The values of absorption and emission bandwidth (*σ*_abs_ and *σ*_emi_) in full width half maximum (FWHM) were extracted from the absorption and emission spectra reported in the published paper when they were not directly reported. Therefore, the error is much smaller than the thickness of the linewidth of spectra. The experimental uncertainty of *σ*_abs_ and *σ*_emi_ in FWHM is estimated to be a maximum of 2 nm.

#### Experimental uncertainty of Φ_QY_

The photoluminescence quantum yield (Φ_QY_**)** is found to be the most error-prone quantity among seven optical properties. The experimental error in Φ_QY_ is affected by several factors such as experimental instruments, measuring methods (absolute *vs* relative), and molecular oxygen (O_2_). The IUPAC technical report is useful for estimating the error in Φ_QY_^17^. The Φ_QY_ of 9,10-diphenylanthracene in cyclohexane is in the range of 0.9 to 0.97. Based on the fact that Φ_QY_ is error-prone, the experimental uncertainty in Φ_QY_ is conservatively estimated to be a maximum of 0.1.

#### Experimental uncertainty of τ_flu_

The fluorescence lifetime (*τ*_flu_) is determined by an exponential fit to the time-resolved fluorescence (TRF) signal. The experimental uncertainty of *τ*_flu_ results mainly from the instrument response function (IRF) and multi-exponential fit process. Since the IRF determines the time-resolution of the TRF spectrometer, the experimental error of *τ*_flu_ is significant when *τ*_flu_ is shorter than the IRF. In most cases. the multi-exponential fitting error does not exceed a maximum of 1%. We collected *τ*_flu_ that was substantially larger than the IRF. Therefore, the experimental uncertainty of *τ*_flu_ is conservatively estimated to be 1%.

#### Experimental uncertainty of log_10_(ε_max_)

To determine the extinction coefficient (*ε*_max_), the absorbance (*A*) and the concentration (*c*) of chromophores should be known based on the Beer’s law (*A* = *εbc* where *b* is the pathlength). Considering that the published papers are peer-reviewed, the experimental error in the concentration is assumed to be less than 5%. Therefore, the experimental uncertainty of log_10_(*ε*_max_) is estimated to be less than 0.02 which is corresponding to log_10_(1.05).

## Data Availability

The optical properties of the chromophores were extracted from the scientific literatures, which is available at 10.6084/m9.figshare.12045567.v2^12^. We have opened a user-friendly webpage (http://Deep4Chem.korea.ac.kr/search) where users can search for chromophores in the database. The database of this webpage will be updated regularly.
